# The Association of Endothelin-1 with Markers of Arterial Stiffness in Black South African Women: The SABPA Study

**DOI:** 10.1155/2015/481517

**Published:** 2015-12-28

**Authors:** Christine Susara du Plooy, Catharina Martha Cornelia Mels, Hugo Willem Huisman, Ruan Kruger

**Affiliations:** ^1^Hypertension in Africa Research Team (HART), North-West University, Potchefstroom 2531, South Africa; ^2^Medical Research Council, Research Unit for Hypertension and Cardiovascular Disease, Faculty of Health Sciences, North-West University, Potchefstroom, South Africa

## Abstract

*Background*. Limited data exist regarding endothelin-1 (ET-1), a vasoactive contributor in vascular tone, in a population subjected to early vascular deterioration. We compared ET-1 levels and explored its association with markers of arterial stiffness in black and white South Africans.* Methodology*. This cross-sectional substudy included 195 black (men: *n* = 99; women: *n* = 95) and 197 white (men: *n* = 99; women: *n* = 98) South Africans. Serum ET-1 levels were measured as well as markers of arterial stiffness (blood pressure, pulse wave velocity, and arterial compliance). ET-1 levels were higher in black men and white women compared to their counterparts after adjusting for C-reactive protein. In both single and partial (adjusting for body mass index and gamma glutamyl transferase) regression analyses ET-1 correlated with age, interleukin-6, high density lipoprotein cholesterol, systolic blood pressure, pulse pressure, and pulse wave velocity in black women. In multivariate regression analyses the independent association of ET-1 with systolic blood pressure (Adj. *R* ^2^ = 0.13; *β* = 0.28, *p* < 0.01) and pulse pressure (Adj. *R* ^2^ = 0.11; *β* = 0.27, *p* < 0.01) was confirmed in black women only. ET-1 additionally associated with interleukin-6 in black women (*p* < 0.01).* Conclusion*. Our result suggests that ET-1 and its link with subclinical arteriosclerosis are potentially driven by low-grade inflammation as depicted by the association with interleukin-6 in the black female cohort.

## 1. Introduction

The high incidence of hypertension and development of early vascular deterioration among the black population remains to be elucidated [1–3]. Vascular aging is characterised by vascular dysfunction manifested as thinning, fraying, and fracturing of elastic laminae and increasing connective tissue and collagen fibers that will lead to stiffening of the arteries [4, 5]. Apart from natural or biological ageing, it is also known that hypertension, atherosclerosis, type 2 diabetes mellitus, chronic renal disease, excessive salt use, and changes in neurohormonal regulation influence the onset and augmentation of arterial stiffness [6].

ET-1 is an important vasoactive biomarker due to its pivotal role in vascular tone and dysfunction [7, 8]. Endothelial dysfunction is a precursor vascular disease usually elicited by the release of a variety of paracrine factors such as endothelin-1 that interact with platelets, inflammatory cells, and the vessel wall [9]. Experimental studies demonstrated that higher ET-1 levels associated with aging may contribute to vascular endothelial dysfunction [10–13]. ET-1 was also positively associated with large artery stiffness in patients with coronary artery disease [14, 15]. The upregulation of ET-1 activates inflammatory cells and leads to nitric oxide synthase inhibition associated with arterial stiffness [7, 9, 16–18].

Ergul et al. [19–21] reported that the black population have higher ET-1 levels and disparities in renal and smooth muscle cell endothelin receptors and are also predisposed to early vascular alterations compared to white individuals. However, limited information is available on the link between ET-1 and arterial stiffness (or arteriosclerosis), especially in sub-Saharan Africa, and therefore we aimed to compare ET-1 levels and explore the association of ET-1 with markers of arterial stiffness along with its potential determinants in a black and white South African cohort.

## 2. Materials and Methods

### 2.1. Study Population and Protocol

Cross-sectional data from the Sympathetic Activity and Ambulatory Blood Pressure in Africans (SABPA) study was used including 194 black (men: *n* = 99; women: *n* = 95) and 197 white (men: *n* = 99; women: *n* = 98) South Africans. Detailed information regarding the procedures of the SABPA study has been published previously [22]. Patients who were pregnant, were lactating, and were using alpha and beta blockers and patients with an ear temperature ≥37°C and who had a vaccination or donated blood 3 months prior to participation were excluded from the prospective cohort study. However, in the current analysis we excluded outliers of ET-1 (*n* = 10) by residual statistics with participants with missing ET-1 data (*n* = 6). A standard health survey was used for the collection of demographic information and antihypertension medication usage. The Health Research Ethics Committee of the North-West University, Potchefstroom Campus, granted ethical approval for this substudy (NWU-00036-07-A6). The study protocol conforms to the ethical guidelines of the Declaration of Helsinki (as revised in 2008) for the investigation on human subjects.

### 2.2. Anthropometric Measurements

Body composition of each participant was obtained in triplicate according to standard procedures [23]. The body height was measured to the nearest 1.0 cm (Invicta Plastics 1465, Leicester, UK) and body mass to the nearest 0.1 kg (Precision Health Scale, A&D Company, Tokyo, Japan). Waist circumference was measured with a nonstretchable metal flexible measuring tape (Holtain Ltd., Dyfed, UK) and body mass index rounded to 1 decimal point [24].

### 2.3. Cardiovascular Measurements

The cardiovascular measurements were taken in a semirecumbent position for each participant. Five-minute continuous measurements of cardiovascular variables were recorded using the validated Finometer (Finapres Medical Systems, Amsterdam, Netherlands), based on the vascular unloading technique of Peñáz, and were processed with the Beatscope 1.1 software to obtain systolic blood pressure, diastolic blood pressure, mean arterial pressure, pulse pressure, stroke volume, and arterial compliance [25]. The Complior SP Acquisition System (Artech-Medical, Pantin, France) was used to measure pulse wave velocity from the carotid to dorsalis pedis.

### 2.4. Biochemical Analyses

A fasting blood sample was collected from each participant and serum prepared according to standard procedures. Serum samples were frozen at −80°C until analysed. ET-1 was determined by an ET-1 Quantikine enzyme linked immunosorbent assay (ELISA) (R&D Systems, Minneapolis, MN, USA). Intra- and interassay variability for ET-1 were 2.7% and 17.15%, respectively. Serum interleukin-6 was determined with a high sensitivity interleukin-6 Quantikine ELISA (R&D Systems, Minneapolis, MN, USA). Intra- and interassay variation of interleukin-6 were 4.2% and 6.4%, respectively. Serum cotinine was determined with a homogenous immunoassay on a Roche Modular system (Roche, Basil, Switzerland). Fasting lipids (triglycerides, total and high density lipoprotein cholesterol), glycated hemoglobin A1c, C-reactive protein, and gamma glutamyl transferase were determined using two sequential multiple analysers in serum samples (Konelab 20i, Thermo Scientific, Vantaa, Finland, and Unicel DXC 800, Beckman and Coulter, Germany). Intra- and interassay variability were less than 10%. Gamma glutamyl transferase was used as an indication of alcohol abuse and cotinine for smoking [26, 27]. Low density lipoprotein cholesterol was calculated with the Friedewald formula: low density lipoprotein cholesterol = total cholesterol – high density lipoprotein cholesterol – (triglycerides/2.2), provided that no value of triglycerides inserted is higher than 4000 mmol/L [28]. Urinary albumin and creatinine levels were determined (Konelab 20i, Thermo Scientific, Vantaa, Finland; Unicel DXC 800, Beckman and Coulter, Germany) and the albumin : creatinine ratio was calculated [29]. Estradiol levels were determined using an electrochemiluminescence immunoassay (ECLIA) (Elecsys 2010, Roche, Basil, Switzerland). Intra- and interassay variability were less than 10% for both albumin : creatinine ratio and estradiol.

### 2.5. Statistical Analyses

G^*∗*^Power version 3.1.9.2 software was used to compute the achieved power in post hoc analysis to determine a fixed model, single regression coefficient for black women in linear regression analysis [30]. At *α* error probability of 0.05, effect size (f2) of 0.15, and one-tailed input method, the achieved power (1-*β* error probability) was estimated at 98.21%. Statistical analyses were done using IBM SPSS Statistics version 22 (IBM Corp., Armonk, NY, USA, 2013). Main effects of race and sex were tested on the associations between ET-1 and cardiovascular components by means of multiple regression.* t*-tests were used to compare means and Chi-square tests to compare proportions between the groups. Bivariate and partial correlations were used to determine the correlation of ET-1 with cardiovascular and biochemical variables. Forward stepwise multiple regression analyses were performed to determine independent associations between ET-1 and cardiovascular measures. We applied a sensitivity analysis for estradiol and antihypertensive medication as covariates in the same multiple regression models.

**Figure 1:**
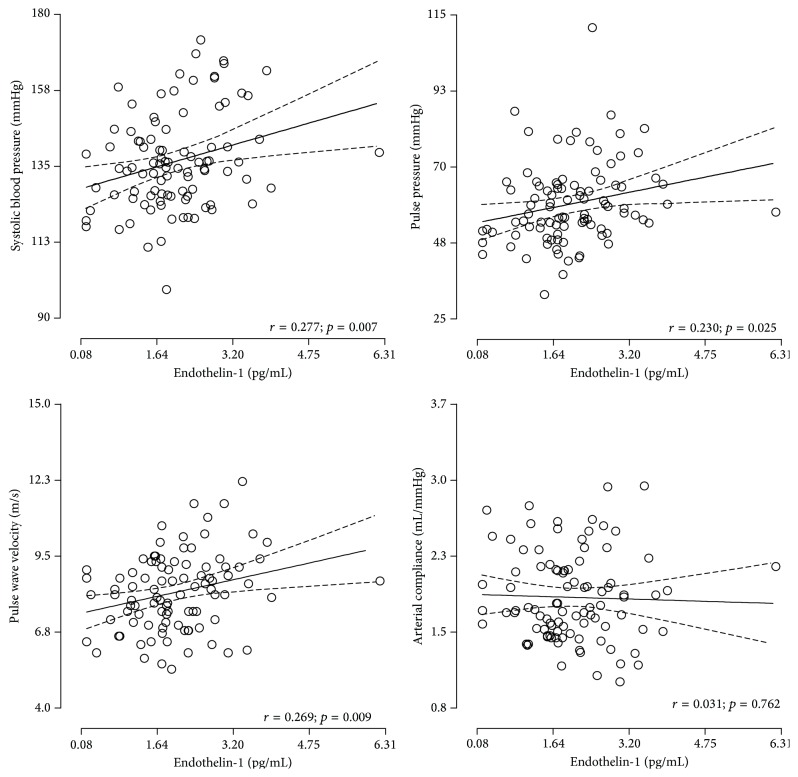
Endothelin-1 with systolic blood pressure, pulse pressure, pulse wave velocity, and arterial compliance in black women only.

The following covariates were considered for entry into the models: age, waist circumference, gamma glutamyl transferase, glycated hemoglobin A1c, high density lipoprotein cholesterol, albumin : creatinine ratio, and interleukin-6. Each model included a main independent measure of vascular function or arterial stiffness, that is, model 1 with systolic blood pressure, model 2 with pulse pressure, model 3 with arterial compliance, and model 4 with pulse wave velocity, whereas ET-1 was the designated dependent variable. Additionally in model 3 (pulse wave velocity) mean arterial pressure was added to the list of covariates. Graphpad v5.03 (GraphPad Software, Inc., San Diego, California, USA) was used to plot endothelin-1 against systolic blood pressure, pulse pressure, pulse wave velocity, and arterial compliance in black women only (Figure 1).

## 3. Results

Basic descriptive characteristics of this study population are listed in Table 1. Due to significant interactions on the association of ET-1 with systolic blood pressure, the population was stratified according to race (*F*(391) = 6.78; *p* < 0.001) and sex (*F*(391) = 2.39; *p* < 0.05).

There was no significant difference in ET-1 levels between the black and white groups before adjusting for C-reactive protein. We additionally adjusted for C-reactive protein to investigate the effect of inflammation on ET-1 levels. After adjusting for C-reactive protein, the ET-1 levels were significantly higher in black men than white men and higher in white women than black women (all *p* < 0.001). Black men and women had higher systolic blood pressure, diastolic blood pressure, mean arterial pressure, and pulse wave velocity in comparison with their white counterparts (all *p* < 0.05). C-reactive protein, interleukin-6, and glycated hemoglobin A1c were higher in the black compared to the white groups (all *p* < 0.05). White participants had higher low density lipoprotein cholesterol and total cholesterol levels in comparison with black individuals (*p* < 0.001). Gamma glutamyl transferase levels were higher in black men and women (*p* < 0.001), with no significant difference in cotinine levels between the two groups.

In bivariate analysis (see Supplementary Table  1 in Supplementary Material available online at http://dx.doi.org/10.1155/2015/481517), no correlations were evident between ET-1 and cardiovascular or biochemical variables in men. A positive correlation was observed between ET-1 and systolic blood pressure (*r* = 0.27; *p* = 0.008), pulse pressure (*r* = 0.25; *p* = 0.014), and pulse wave velocity (*r* = 0.23; *p* = 0.026) in black women only (Figure 1). In white women a positive correlation was observed between ET-1 and stoke volume (*r* = 0.23; *p* = 0.026). A positive correlation was observed between ET-1 and age (*r* = 0.26; *p* = 0.009), interleukin-6 (*r* = 0.27; *p* = 0.007), high density lipoprotein cholesterol (*r* = 0.23; *p* = 0.026), and a borderline positive correlation with mean arterial pressure (*r* = 0.20; *p* = 0.053) in black women.

After adjustments for body mass index and gamma glutamyl transferase (Table 2), the positive correlation remained between ET-1 and interleukin-6 (*r* = 0.22; *p* = 0.031), high density lipoprotein cholesterol (*r* = 0.25; *p* = 0.017), systolic blood pressure (*r* = 0.26; *p* = 0.013), pulse pressure (*r* = 0.23; *p* = 0.025), pulse wave velocity (*r* = 0.20; *p* = 0.050), and a borderline positive correlation with mean arterial pressure (*r* = 0.19; *p* = 0.063) in the black women. An inverse correlation between ET-1 and arterial compliance (*r* = −0.24; *p* = 0.018) also emerged in black women. The correlation between ET-1 and stroke volume persisted in white women (*r* = 0.24; *p* = 0.021).

Since no significant correlations existed in men, the forward stepwise multiple regression analyses were only performed in women. The previous association between ET-1 and stroke volume in white women disappeared (Adj. *R* ^2^ = 0.025; *β* = 0.12; *p* = 0.55). In black women (Table 3), an independent association of ET-1 with systolic blood pressure (Adj. *R* ^2^ = 0.178; *β* = 0.269; *p* = 0.005), pulse pressure (Adj. *R* ^2^ = 0.159; *β* = 0.233; *p* = 0.017), and mean arterial pressure (Adj. *R* ^2^ = 0.149; *β* = 0.211; *p* = 0.031) was confirmed, but no association with pulse wave velocity (Adj. *R* ^2^ = 0.149; *β* = 0.197; *p* = 0.197) and arterial stiffness (Adj. *R* ^2^ = 0.142; *β* = −0.018; *p* = 0.880) was confirmed. ET-1 also associated with high density lipoprotein cholesterol (all models *p* < 0.05) and interleukin-6 (all models *p* < 0.01) in all four models and with age in model 4 (Adj. *R* ^2^ = 0.142; *β* = 0.199; *p* = 0.047).

### 3.1. Sensitivity Analysis

After performing the same multiple regression analyses and additionally correcting for estrogen and hypertension medication, no change was observed in the previous association of ET-1 with systolic blood pressure (Adj. *R* ^2^ = 0.178; *β* = 0.269; *p* = 0.005), pulse pressure (Adj. *R* ^2^ = 0.159; *β* = 0.233; *p* = 0.017), pulse wave velocity (Adj. *R* ^2^ = 0.149; *β* = 0.197; *p* = 0.197), and arterial compliance (Adj. *R* ^2^ = 0.142; *β* = −0.018; *p* = 0.880) in black women.

## 4. Discussion

It was previously shown that the black population are predisposed to early vascular alterations compared to white individuals Ergul et al. [19–21]; however, limited information is available on the link between ET-1 and arterial stiffness within a biethnic South African population. Our results indicated an independent association of ET-1 with systolic blood pressure and pulse pressure in black women only. We also found an independent association between ET-1 and interleukin-6, suggesting that the link between ET-1 and subclinical vascular dysfunction may be mediated by proinflammation. Our study further contributes to the lack of information regarding ET-1 and cardiovascular function in black populations from sub-Saharan Africa, especially in black women prone to arterial stiffness and hypertensive heart disease.

Levels of ET-1 were not different between race groups in our study population; however after considering C-reactive protein as confounding variable through univariate analysis of covariance, there was a significant difference in ET-1 levels between race groups. White women had higher levels of ET-1 than black women in our population. This is in contradiction with other studies that found that black individuals have higher ET-1 levels than whites [19, 31–33]. The link observed between ET-1 and arterial stiffness in black women with lower ET-1 levels compared to the other groups may suggest that even at this low ET-1 concentrations cardiovascular changes are present and potentially driven by an inflammatory condition as depicted by high C-reactive protein levels and higher prevalence of overweight and obesity. Black men have higher ET-1 levels in our study than white men. This coincides with other studies [19, 31–33]. Since the black population is also subjected to the development of early vascular deterioration, especially arterial stiffness in black women, our result may support this trend in black urbanized women [3]. The lack of association between ET-1 and more pronounced measures of arterial stiffness (pulse wave velocity and arterial compliance) may be due to the younger age of this cohort and these overt changes had not yet occur, apart from higher C-reactive protein levels and marked overweight.

Our study also found a positive association between systolic blood pressure and ET-1 levels, only in black women. Some studies found no difference in ET-1 levels between normotensive and hypertensive participants, whereas other studies suggested a possible link between elevated ET-1 and increasing systolic blood pressure [34–36]. The activation of receptor-bound ET-1 is associated with growth and proinflammatory effects and the remodeling of resistance arteries via increased oxidative stress and consequent vascular endothelial dysfunction [11, 34]. ET_*A*_ receptor activation causes vasoconstriction, enhancement of nerve-stimulated adrenal catecholamine release, and positive inotropy which in turn may increase blood pressure [20, 35, 37] while ET_*B*_ receptor produces vasodilation, increases in sodium excretion, and inhibits growth and inflammation [38]. The black population have both ET_*A*_ and ET_*B*_ receptors on vascular smooth muscle cells, but the total number of ET_*B*_ receptors is lower than that in the white population. Although our study did not assess ET receptors, the decrease of ET_*B*_ receptor ratio on vascular smooth muscle cells previously indicated in black populations [20] could be favoring vasoconstriction-promoting receptors, providing a possible explanation for the association of ET-1 with increased systolic blood pressure [20, 21, 36]. Even though the black men have higher pulse pressure and pulse wave velocity levels than black women, the lack of association of these markers with ET-1 might suggest that black men of this population are already subjected to subclinical organ damage at macrovascular and cardiac level. It is possible that ET-1 could correlate with markers of myocardial damage instead; unfortunately we lack the data to investigate this hypothesis.

Prolonged elevated blood pressure was found to lead to increased ET-1 and interleukin-6 levels [16, 17, 39]. We found higher levels of interleukin-6 and C-reactive protein in the black population compared to the white population and only in black women. ET-1 related positively with interleukin-6. Previous studies have shown that the black population have higher levels of inflammatory markers, especially C-reactive protein and soluble urokinase plasminogen activator receptor compared to the white population [40, 41]. The absence of these findings among white women were discussed by Schutte et al. [41] suggesting that obesity can be associated with chronic activation of the immune system leading to very high levels of inflammatory markers in the black women due to the fact the obesity is an inflammatory condition. Perivascular fat tissue may interact in an autocrine/paracrine manner with the endothelial cells (where ET-1 is released) subsequently contributing to endothelial dysfunction, often associated with markers of inflammation. ET-1 has previously been found to stimulate the release of interleukin-6 and has been implicated in the development of atherosclerosis and vascular dysfunction [7]. ET-1 and interleukin-6 have also been suggested to be involved in the proinflammatory effect of C-reactive protein [7]. Damage or infection to the endothelium causes ET-1 to bind to ET_*B*_ receptors on smooth muscle cells and control the macrophages and its release of inflammatory cytokines such as tumor necrosis factor-alpha, interleukin-6, and interleukin-1 [7, 17, 42, 43]. The aim of our study was not to investigate the role of inflammation and ET-1 in this population group; however we did find a link between interleukin-6 and ET-1. The absence of C-reactive protein associated with ET-1 in the population may be because there is a discrepancy in the adjustments for body composition.

We also observed a positive association between ET-1 and high density lipoprotein cholesterol in our black female group. Previous studies have mentioned that black Africans are prone to hypertension and weight gain, which could lead to elevated high density lipoprotein cholesterol levels [41, 44]. It is noteworthy to mention that the black women in this study population did not suffer from severe arterial stiffness and this may be explained by the protective nature of high density lipoprotein cholesterol having the ability to remove cholesterol from macrophage foam cells in the arterial wall and carry it to the liver for excretion into the bile, reducing the risk to atherosclerosis [45]. The positive association of ET-1 with high density lipoprotein cholesterol may counterregulate proinflammation until arterial stiffness manifests in this group. Although vascular inflammation can be limited by anti-inflammatory counterregulatory mechanisms that maintain the integrity and homeostasis of the vascular wall, chronic exposure to cardiovascular risk factors such as high blood pressure, tobacco overuse, obesity, physical inactivity, and raised blood glucose may render these counterregulatory mechanisms defenseless [18]. Further studies could shed light on the precise mechanism by which ET-1 and inflammation are involved in the development of arterial stiffness (or arteriosclerosis) and subsequent cardiovascular disease in black women.

The results of this study need to be interpreted within the context of its limitations and strengths. This was a cross-sectional study and we cannot pinpoint any cause or effect. Pulse wave velocity was measured in our group; unfortunately femoral pulse wave velocity could not be obtained. Although the results were consistent after multiple adjustments, we cannot exclude residual confounding. Further data on autonomic and endothelial function are needed to delineate possible physiological mechanisms at play. The strength of the study can be measured on the basis that it was a well-designed study under controlled conditions (two ethnic and homogenous socioeconomic groups).

In conclusion, ET-1 independently associated with systolic blood pressure, pulse pressure, and interleukin-6 in black women. Our result suggests that ET-1 and its link with subclinical arteriosclerosis are potentially driven by low-grade inflammation as depicted by interleukin-6 in the black female cohort.

## Supplementary Material

Our study found an unadjusted correlation between endothelin-1 and age, systolic blood pressure, pulse pressure and pulse wave velocity in black women. ET-1 also correlated positively with interleukin-6, high density lipoprotein in black women and stroke volume in white women. No correlations where observed between endothelin-1 and cardiovascular and metabolic markers in black and white men.

## Figures and Tables

**Table 1:** Population characteristics stratified by race and sex.

Variable	Men (*n* = 198)			Women (*n* = 193)		
	Black (*n* = 99)	White (*n* = 99)	*p* value	Black (*n* = 95)	White (*n* = 98)	*p* value
Age (years)	43.1 ± 8.08	45.0 ± 11.1	0.18	45.6 ± 7.95	44.7 ± 10.7	0.52
Body mass index (kg/m^2^)	27.6 ± 5.80	29.1 ± 5.23	0.061	32.9 ± 7.29	26.1 ± 5.62	<0.001
Waist circumference (mm)	93.6 ± 15.5	101.7 ± 14.5	<0.001	93.9 ± 15.6	87.8 ± 13.0	<0.001

Cardiovascular variables						
Systolic blood pressure (mmHg)	146.0 ± 21.0	137.0 ± 13.0	<0.001	136.0 ± 14.0	132.0 ± 15.0	0.042
Diastolic blood pressure (mmHg)	86.0 ± 11.0	80.0 ± 8.0	<0.001	77.0 ± 8.0	73.0 ± 7.00	<0.001
Pulse pressure (mmHg)	61.0 ± 8.0	56.0 ± 7.0	0.018	50.0 ± 10.0	46.0 ± 7.00	0.80
Mean arterial pressure (mmHg)	111.0 ± 14.0	102.0 ± 9.0	<0.001	101.0 ± 9.0	97.0 ± 9.00	<0.001
Stroke volume (mL)	101.0 ± 25.6	103.0 ± 20.4	0.48	102.0 ± 30.0	93.0 ± 24.0	0.014
Arterial compliance (mL/mmHg)	1.89 ± 0.42	2.33 ± 0.52	<0.001	1.86 ± 0.42	1.88 ± 0.40	0.65
Pulse wave velocity (m/s)	9.18 ± 2.29	8.62 ± 1.34	0.039	8.19 ± 1.39	7.47 ± 1.19	<0.001
Hypertension status, *n* (%)	64 (64.6)	85 (85.9)	<0.001	63 (66.3)	87 (88.8)	<0.001
Antihypertensive medication, *n* (%)	35 (17.7)	14 (7.1)	<0.001	33 (16.9)	12 (6.2)	0.019

Biochemical variables						
Endothelin-1 (pg/mL)^*∗*^	2.06 ± 1.67	1.92 ± 1.69	<0.001	1.74 ± 1.87	1.90 ± 1.72	<0.001
C-reactive protein (mg/L)	2.73 (0.27–16.1)	1.82 (0.99–8.20)	0.003	7.09 (0.78–35.7)	2.22 (0.99–14.3)	<0.001
Interleukin-6 (pg/mL)	1.95 (0.33–3.57)	0.87 (0.27–2.90)	0.032	1.24 (0.41–3.06)	0.95 (0.29–3.63)	0.016
Glycated hemoglobin A1c (%)	6.16 (5.20–9.60)	5.65 (5.10–6.60)	0.032	5.79 (5.10–6.60)	5.36 (4.99–5.90)	<0.001
High density lipoprotein cholesterol (mmol/L)	1.04 ± 0.34	1.00 ± 0.27	0.36	1.20 ± 0.31	1.42 ± 0.42	<0.001
Low density lipoprotein cholesterol (mmol/L)	2.86 ± 0.95	3.91 ± 1.07	<0.001	3.05 ± 1.09	3.94 ± 1.11	<0.001
Total cholesterol (mmol/L)	4.72 ± 1.17	5.59 ± 1.21	<0.001	4.46 ± 1.21	5.54 ± 1.31	<0.001
Triglycerides (mmol/L)	1.46 (0.57–4.96)	1.30 (0.54–3.16)	0.16	1.11 (0.42–2.13)	0.81 (0.40–2.22)	0.13
Albumin : creatine ratio (mg/mmol/L)	1.46 ± 1.81	0.40 ± 0.99	<0.001	1.73 ± 3.54	0.80 ± 1.60	0.019
Cotinine (ng/mL)	62.9 (5.00–275.0)	77.3 (1.00–623.0)	0.50	48.4 (3.87–271.1)	91.7 (6.00–307.0)	0.21
Gamma glutamyl transferase (U/L)	63.0 (23.6–382.9)	27.5 (11.0–101.9)	<0.001	35.6 (16.7–116.6)	14.2 (6.00–41.0)	<0.001

Values are arithmetic mean ± standard deviation, geometric mean (5th and 95th confidence interval), or number of participants. ^*∗*^Analysis of covariance was performed by adjusting for C-reactive protein only.

**Table 2:** Partial correlations of endothelin-1 with cardiometabolic variables adjusted for body mass index and gamma glutamyl transferase.

	Endothelin-1 (pg/mL)			
	Men (*n* = 198)		Women (*n* = 193)	
	Black (*n* = 99)	White (*n* = 99)	Black (*n* = 95)	White (*n* = 98)
Cardiovascular variables				
Systolic blood pressure (mmHg)	*r* = 0.097; *p* = 0.35	*r* = 0.078; *p* = 0.45	*r* = 0.26; *p* = 0.013	*r* = 0.12; *p* = 0.23
Diastolic blood pressure (mmHg)	*r* = 0.028; *p* = 0.79	*r* = 0.15; *p* = 0.16	*r* = 0.11; *p* = 0.27	*r* = −0.047; *p* = 0.65
Pulse pressure (mmHg)	*r* = 0.11; *p* = 0.28	*r* = −0.022; *p* = 0.83	*r* = 0.23; *p* = 0.025	*r* = 0.18; *p* = 0.080
Heart rate (beats per minute)	*r* = −0.050; *p* = 0.64	*r* = −0.002; *p* = 0.99	*r* = 0.16; *p* = 0.13	*r* = −0.11; *p* = 0.29
Mean arterial pressure (mmHg)	*r* = 0.061; *p* = 0.56	*r* = 0.105; *p* = 0.31	*r* = 0.19; *p* = 0.063	*r* = 0.048; *p* = 0.64
Stroke volume (mL)	*r* = 0.12; *p* = 0.26	*r* = −0.103; *p* = 0.31	*r* = −0.001; *p* = 0.99	*r* = 0.24; *p* = 0.021
Arterial compliance (mL/mmHg)	*r* = 0.012; *p* = 0.91	*r* = −0.064; *p* = 0.53	*r* = −0.24; *p* = 0.018	*r* = 0.11; *p* = 0.29
Pulse wave velocity (m/s)	*r* = 0.13; *p* = 0.21	*r* = 0.15; *p* = 0.14	*r* = 0.20; *p* = 0.050	*r* = 0.11; *p* = 0.29

Biochemical variables				
Interleukin-6 (pg/mL)	*r* = −0.031; *p* = 0.76	*r* = −0.15; *p* = 0.14	*r* = 0.22; *p* = 0.031	*r* = −0.14; *p* = 0.16
Glycated hemoglobin A1c (%)	*r* = −0.047; *p* = 0.65	*r* = 0.004; *p* = 0.97	*r* = 0.12; *p* = 0.27	*r* = 0.007; *p* = 0.95
Total cholesterol (mmol/L)	*r* = 0.064; *p* = 0.53	*r* = 0.050; *p* = 0.62	*r* = 0.044; *p* = 0.67	*r* = −0.070; *p* = 0.50
High density lipoprotein cholesterol (mmol/L)	*r* = 0.050; *p* = 0.63	*r* = 0.16; *p* = 0.12	*r* = 0.25; *p* = 0.017	*r* = 0.060; *p* = 0.56
Triglycerides (mmol/L)	*r* = 0.077; *p* = 0.46	*r* = 0.081; *p* = 0.43	*r* = −0.020; *p* = 0.85	*r* = −0.023; *p* = 0.82
Albumin : creatinine ratio (mg/mmol/L)	*r* = 0.058; *p* = 0.57	*r* = −0.065; *p* = 0.53	*r* = 0.042; *p* = 0.68	*r* = 0.087; *p* = 0.40

**Table 3:** Forward stepwise regression analyses of endothelin-1 with measures of arterial stiffness in black women.

	Endothelin-1 (pg/mL) (*n* = 95)	
Model 1: systolic blood pressure	Adj. *R* ^2^ = 0.178	
	Std. *β* (95% CI)	*p* value

Systolic blood pressure (mmHg)	0.269 (0.083–0.455)	0.005
Interleukin-6 (pg/mL)	0.290 (0.104–0.476)	0.003
High density lipoprotein cholesterol (mmol/L)	0.203 (0.047–0.419)	0.016

Model 2: pulse pressure	Adj. *R* ^2^ = 0.159	
	Std. *β* (95% CI)	*p* value

Pulse pressure (mmHg)	0.233 (0.045–0.421)	0.017
Interleukin-6 (pg/mL)	0.278 (0.090–0.466)	0.005
High density lipoprotein cholesterol (mmol/L)	0.231 (0.043–0.419)	0.018

Model 3: pulse wave velocity	Adj. *R* ^2^ = 0.149	
	Std. *β* (95% CI)	*p* value

Interleukin-6 (pg/mL)	0.296 (0.108–0.484)	0.003
High density lipoprotein cholesterol (mmol/L)	0.239 (0.051–0.427)	0.015
Mean arterial pressure	0.211 (0.023–0.399)	0.031

Model 4: arterial compliance	Adj. *R* ^2^ = 0.142	
	Std. *β* (95% CI)	*p* value

Interleukin-6 (pg/mL)	0.258 (0.066–0.450)	0.010
Age (years)	0.199 (0.005–0.393)	0.047
High density lipoprotein cholesterol (mmol/L)	0.209 (0.017–0.401)	0.036

Covariates considered for entry into the models included age, waist circumference, gamma glutamyl transferase, glycated hemoglobin A1c, high density lipoprotein, albumin : creatinine ratio, total cholesterol, interleukin-6, and in model 3 additionally mean arterial pressure.
