# Supplementary material for: The Association of Endothelin-1 with Markers of Arterial Stiffness in Black South African Women: The SABPA Study
Source: J Amino Acids. 2015 Dec 28;2015:481517. doi: 10.1155/2015/481517 (PMC4707353; doi:10.1155/2015/481517)
Supplement: Supplementary file 1 — Our study found an unadjusted correlation between endothelin-1 and age, systolic blood pressure, pulse pressure and pulse wave velocity in black women. ET-1 also correlated positively with interleukin-6, high density lipoprotein in black women and stroke volume in white women. No correlations where observed between endothelin-1 and cardiovascular and metabolic markers in black and white men. [file 481517.f1.pdf]

SUPPLEMENTAL MATERIAL

Supplementary Table 1. Single correlations of endothelin-1 with cardiovascular and metabolic markers in black and white men and women.

	**Endothelin-1 (pg/mL)**			
	**Men (n = 198)**		**Women (n = 193)**	
	**Black (n = 99)**	**White (n = 99)**	**Black (n = 95)**	**White (n = 98)**
Age (years)	r=0.041; p=0.69	r=–0.104; p=0.30	r=0.26; p=0.009	r=–0.006; p=0.96
Body mass index (kg/m^2)	r=0.030; p=0.77	r=0.065; p=0.52	r=0.17; p=0.093	r=0.056; p=0.58
Waist circumference (mm)	r=0.055; p=0.59	r=0.096; p=0.34	r=0.14; p=0.17	r=0.051; p=0.62
**Cardiovascular variables**				
Systolic blood pressure (mmHg)	r=0.12; p=0.24	r=0.049; p=0.63	r=0.27; p=0.008	r=0.14; p=0.18
Diastolic blood pressure (mmHg)	r=0.048; p=0.65	r=0.153; p=0.13	r=0.12; p=0.27	r=-0.024; p=0.82
Pulse pressure (mmHg)	r=0.14; p=0.19	r=–0.069; p=0.50	r=0.25; p=0.014	r=0.19; p=0.063
Mean arterial pressure (mmHg)	r=0.087; p=0.40	r=0.089; p=0.38	r=0.20; p=0.053	r=0.065; p=0.52
Stroke volume (mL)	r=0.13; p=0.22	r=–0.093; p=0.36	r=0.11; p=0.30	r=0.23; p=0.026
Arterial compliance (mL/mmHg)	r=–0.008; p=0.94	r=–0.011; p=0.91	r=–0.078; p=0.45	r=0.13; p=0.22
Pulse wave velocity (m/s)	r=0.15; p=0.15	r=0.118; p=0.25	r=0.23; p=0.026	r=0.11; p=0.27
**Biochemical analyses**				
C-Reactive Protein (mg/L)	r=0.015; p=0.88	r=0.178; p=0.078	r=0.090; p=0.38	r=–0.016; p=0.87
Interleukin-6 (pg/mL)	r=–0.017; p=0.87	r=–0.139; p=0.17	r=0.27; p=0.007	r=–0.118; p=0.25
Glycated hemoglobin A1c (%)	r=–0.024; p=0.81	r=0.020; p=0.84	r=0.16; p=0.13	r=0.010; p=0.92
High density lipoprotein cholesterol (mmol/L)	r=0.040; p=0.69	r=0.137; p=0.18	r=0.23; p=0.026	r=0.041; p=0.69
Low density lipoprotein cholesterol (mmol/L)	r=0.025; p=0.81	r=–0.040; p=0.69	r=0.011; p=0.91	r=–0.090; p=0.38
Total cholesterol (mmol/L)	r=0.100; p=0.32	r=0.004; p=0.97	r=0.082; p=0.43	r=–0.059; p=0.56
Triglycerides (mmol/L)	r=0.155; p=0.12	r=–0.010; p=0.92	r=0.030; p=0.77	r=0.002; p=0.98
Albumin-to-creatinine ratio (mg/mmol/L)	r=0.040; p=0.70	r=–0.069; p=0.50	r=0.046; p=0.66	r=0.093; p=0.36
Cotinine (ng/mL)	r=–0.17; p=0.32	r=0.044; p=0.87	r=0.28; p=0.24	r=0.65; p=0.41
Gamma glutamyl transferase (U/L)	r=0.18; p=0.075	r=–0.170; p=0.092	r=0.15; p=0.15	r=–0.018; p=0.86
